# Economic Evaluation of First-Line Adjuvant Chemotherapies for Resectable Gastric Cancer Patients in China

**DOI:** 10.1371/journal.pone.0083396

**Published:** 2013-12-10

**Authors:** Chongqing Tan, Liubao Peng, Xiaohui Zeng, Jianhe Li, Xiaomin Wan, Gannong Chen, Lidan Yi, Xia Luo, Ziying Zhao

**Affiliations:** 1 Department of Pharmacy, The Second Xiangya Hospital of Central South University, Changsha, People’s Republic of China; 2 School of Pharmaceutical Sciences, Central South University, Changsha, Hunan, People’s Republic of China; 3 PET-CT center, The Second Xiangya Hospital of Central South University, Changsha, People’s Republic of China; 4 Department of Surgery, The Second Xiangya Hospital of Central South University, Changsha, People’s Republic of China; Faculty of Medicine, University of Porto, Portugal

## Abstract

**Background:**

First-line postoperative adjuvant chemotherapies with S-1 and capecitabine and oxaliplatin (XELOX) were first recommended for resectable gastric cancer patients in the 2010 and 2011 Chinese NCCN Clinical Practice Guidelines in Oncology: Gastric Cancer; however, their economic impact in China is unknown.

**Objective:**

The aim of this study was to compare the cost-effectiveness of adjuvant chemotherapy with XELOX, with S-1 and no treatment after a gastrectomy with extended (D2) lymph-node dissection among patients with stage II-IIIB gastric cancer.

**Methods:**

A Markov model, based on data from two clinical phase III trials, was developed to analyse the cost-effectiveness of patients in the XELOX group, S-1 group and surgery only (SO) group. The costs were estimated from the perspective of Chinese healthcare system. The utilities were assumed on the basis of previously published reports. Costs, quality-adjusted life-years (QALYs) and incremental cost-effectiveness ratios (ICER) were calculated with a lifetime horizon. One-way and probabilistic sensitivity analyses were performed.

**Results:**

For the base case, XELOX had the lowest total cost ($44,568) and cost-effectiveness ratio ($7,360/QALY). The relative scenario analyses showed that SO was dominated by XELOX and the ICERs of S-1 was $58,843/QALY compared with XELOX. The one-way sensitivity analysis showed that the most influential parameter was the utility of disease-free survival. The probabilistic sensitivity analysis predicted a 75.8% likelihood that the ICER for XELOX would be less than $13,527 compared with S-1. When ICER was more than $38,000, the likelihood of cost-effectiveness achieved by S-1 group was greater than 50%.

**Conclusions:**

Our results suggest that for patients in China with resectable disease, first-line adjuvant chemotherapy with XELOX after a D2 gastrectomy is a best option comparing with S-1 and SO in view of our current study. In addition, S-1 might be a better choice, especially with a higher value of willingness-to-pay threshold.

## Introduction

Stomach cancer is the fourth most common malignancy in the world, and 46.9% (463,000 cases) of the cases occur in China. Approximately 352,000 people die from gastric cancer in China each year [1]. Adjuvant chemotherapy, which is a standard component of treatment after surgery, can improve overall survival (OS) and progression-free survival (PFS) for resectable gastric cancer patients [2,3]. A strategy that includes S-1, new adjuvant chemotherapy after gastrectomy with extended (D2) lymph-node dissection, was recommended in the 2010 Chinese NCCN Clinical Practice Guidelines in Oncology: Gastric Cancer [4]. The capecitabine and oxaliplatin (XELOX) regimen was added in the 2011 guidelines [5]. 

S-1 (TS-1, Taiho Pharmaceutical) is an oral anticancer treatment that combines tegafur (a prodrug that is metabolised to fluorouracil, largely by P-450 enzymes in the liver), gimeracil (an inhibitor that prevents the degradation of fluorouracil) and oteracil (an inhibitor that prevents the phosphorylation of fluorouracil in the gastrointestinal tract). S-1 is an active anticancer agent against advanced gastric cancer when administered alone or in combination with other chemotherapies, and its effectiveness has been demonstrated in several trials [6-9]. ACTS-GC (registered at ClinicalTrials.gov, NCT00152217), a multicentre randomised controlled trial, showed that postoperative adjuvant therapy with S-1 can improve overall survival and relapse-free survival for East Asian patients who have undergone a D2 gastrectomy for stage II-IIIB gastric cancer [10]. 

Capecitabine is an oral prodrug of fluoropyrimidine that is converted to fluorouracil in tumour tissue in a reaction that is catalysed by the enzyme thymidine phosphorylase [11]. Oxaliplatin is a cisplatin derivative that forms bulky platinum-DNA adducts to restrain DNA synthesis and repair [12]. Several phase II trials have shown that oral capecitabine plus intravenous oxaliplatin was effective and well-tolerated as a first-line chemotherapy regimen for patients with advanced gastric cancer [13-15]. The CLASSIC (registered at ClinicalTrials.gov, NCT00411229) trial showed a survival benefit in patients who received the regimen of adjuvant capecitabine plus oxaliplatin after curative D2 gastrectomy for stage II-IIIB gastric cancer [16].

In China, capecitabine (Xeloda; Roche, Shanghai, China) costs $6.3 for 500 mg, oxaliplatin (Eloxatin; Sanofi-Aventis, Hangzhou, China) costs $413.6 for 50 mg, and S-1 (TS-1, Taiho Pharmaceutical, Japan) costs $9.64 for 20mg [17]. Patients with resectable gastric cancer in China have three treatment choices after surgery: no treatment (i.e., surgery only), adjuvant chemotherapy with XELOX, and adjuvant chemotherapy with S-1. It is of great importance to consider the cost-effectiveness of the treatments for decision-makers, especially in resource-limited country, such as China. 

The aim of this study, from the perspective of Chinese healthcare system, was to compare cost-effectiveness of two first-line adjuvant chemotherapy regimens and D2 gastrectomy alone for patients with stage II-IIIB gastric cancer, based on the clinical results of the CLASSIC and ACTS-GC trials.

## Methods

### Economic model

**Figure 1:**
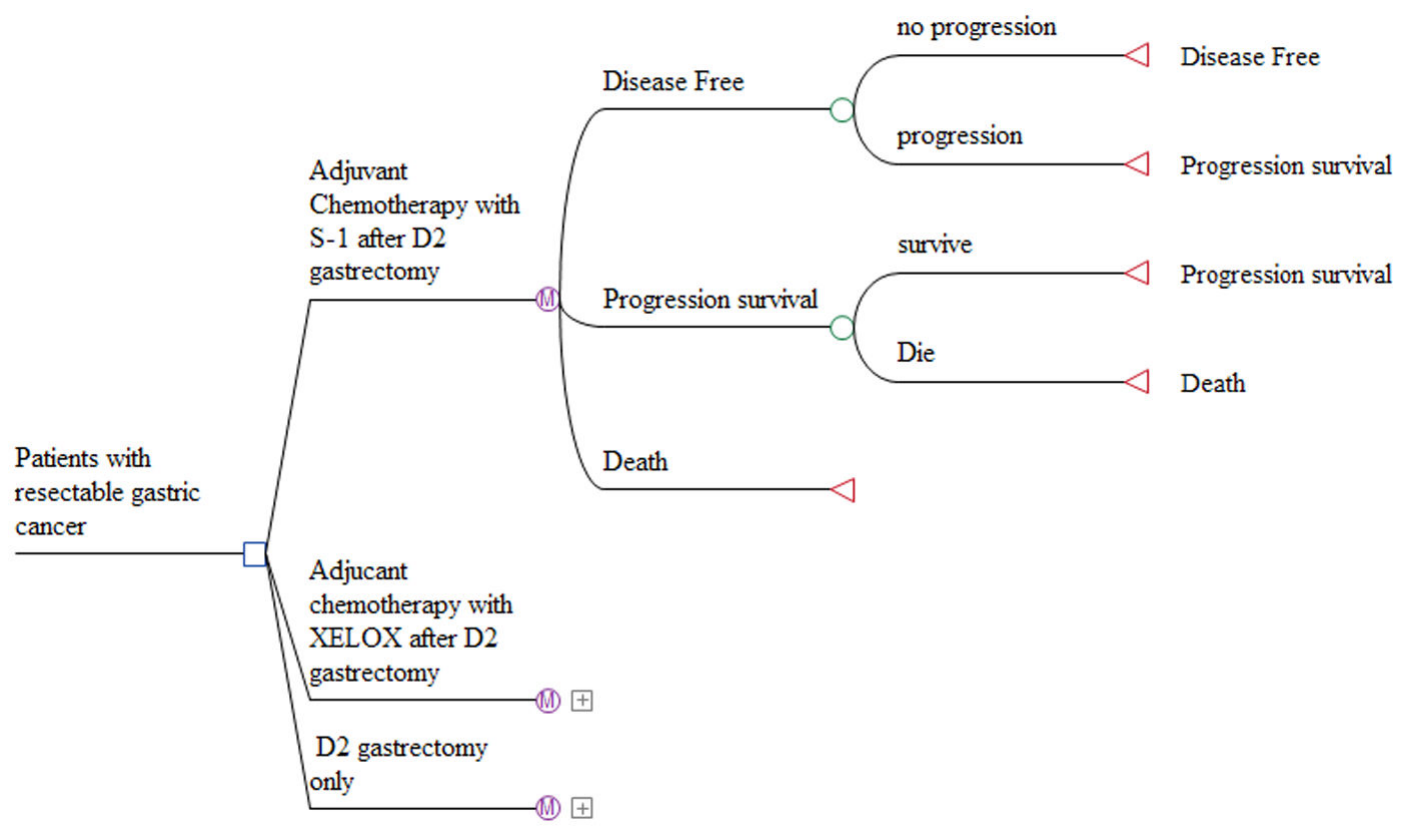
Markov model used to evaluate three treatment strategies for resectable gastric cancer.

No clinical trials have directly compared the XELOX, S-1 and surgery-only (SO) strategies; however, there were striking parallels in the study design, eligibility criteria and control group composition of the CLASSIC and ACTS-GC trials. Both clinical trials were designed in East Asia for patients with stage II-IIIB gastric cancer. In the trials, 2094 patients received a D2 gastrectomy and achieved no cancer at resection margins. In the CLASSIC trial, 1035 patients were randomly assigned to receive 168 days of XELOX or no treatment after surgery. The PFS of XELOX group was 74%, compared with 59% in the SO group (HR 0.56). In the ACTS-GC trial, 1059 patients were randomly assigned to receive 1 year of oral S-1 or no treatment after surgery; the PFS of S-1 group and SO group were 80.1% and 70.1% (HR 0.68). The brief information of the two trials was shown in Table 1. A Markov model, based on the two trials, was constructed using TreeAge Pro 2011 software (Figure 1). This model was designed to evaluate the economic and clinical data of the patients with stage II-IIIB gastric cancer in the XELOX group, the S-1 group and the SO group. Every group included patients in three mutually exclusive health states: disease-free survival (DFS), progression survival (PS) and death. Our model was performed to track with a lifetime horizon, with the cycle of 6 weeks. All of the patients were assumed to be 60 years old and progression-free at stage 0. The annual discount rate for costs and utilities was set at 3%, according to the requirement of China Guidelines for Pharmacoeconomic Evaluations [18]. The willingness-to-pay (WTP) threshold was assumed to be three times the Chinese per capita GDP.

**Table 1:** Compared with CLASSIC and ACTS-GC Trials.

	**CLASSIC Trial[16]**		**ACTS-GC Trial[3]**	
**Design of trial**	Randomized, open-label, multicenter, parallel-group		Randomized, open-label, multicenter, parallel-group	
Centres	In South Korea, China, and Taiwan		In Japan	
**Eligible criteria**				
Age	>18		20-80	
Histologically confirmed	stageⅡ, ⅢA, ⅢB		stageⅡ, ⅢA, ⅢB	
Surgery	had D2 surgery and achieved R0 resection		had D2 surgery and achieved R0 resection	
**Procedures**				
Control group	D2 gastrectomy only		D2 gastrectomy only	
Trial group	eight 3-week cycles of oral capecitabine (1000mg/m^2^, twice a day, on days 1-14 of each cycle) plus intravenous oxaliplatin (130mg/m^2^, once a day , on day 1 of each cycle) after surgery		1 year 6-week cycles of oral S-1 (40mg/m^2^, twice a day, on days 1-28 of each cycle) after surgery	
**Results**	**Trial group**	**Control group**	**Trial group**	**Control group**
Patients	520	515	529	519
Age (years)	56.1	55.8	63	63
3 years PFS	0.74	0.59	0.722	0.596
HR of 3 years PFS	0.56		0.62	
3 years OS	0.83	0.78	0.801	0.701
HR of 3 years OS	0.72		0.68	

PFS = progress-free survival; OS = overall survival; HR = hazard ratio

The transition probabilities were obtained from the two trials. Using R software (Version 2.15.1), we assumed an average survival rate of SO group (reference) and simulated Weibull survival curves based on the data extracted from the Kaplan-Meier curves of the SO group in the two trials. Meanwhile, a statistical test was provided by Akaike’s Information Criterion (AIC) and Bayesian Information Criterion (BIC) to examine the goodness of fit by Exponential or Weibull distribution (Table 2) [19]. In addition, the validity of the simulated survival curves tail beyond 3 years was conducted by comparing the 5 years overall survival rate calculated from possible distribution models (Exponential: 61.1% or Weibull: 62.6%) to the data from the Surveillance, Epidemiology and End Results (SEER) Program, which shows that 5 year overall survival rate of localized gastric cancer patients is 63.2% [20]. Therefore, on the basis of two points above, Weibull distribution is more suitable than Exponential distribution.

**Table 2:** Goodness of fit by the possible distributions of survival curves for surgery-only group.

**Distribution**	**PFS**				**OS**		
	**Adjusted R^2^**	**AIC**	**BIC**		**Adjusted R^2^**	**AIC**	**BIC**
Exponential	0.9853	-221.674	-220.378		0.9695	-223.471	-222.175
Weilbull	0.9866	-222.152	-219.560		0.9907	-253.445	-250.854

PFS = progress-free survival; OS = overall survival; AIC = Akaike’s Information Criterion; BIC = Bayesian Information Criterion.

**Figure 2:**
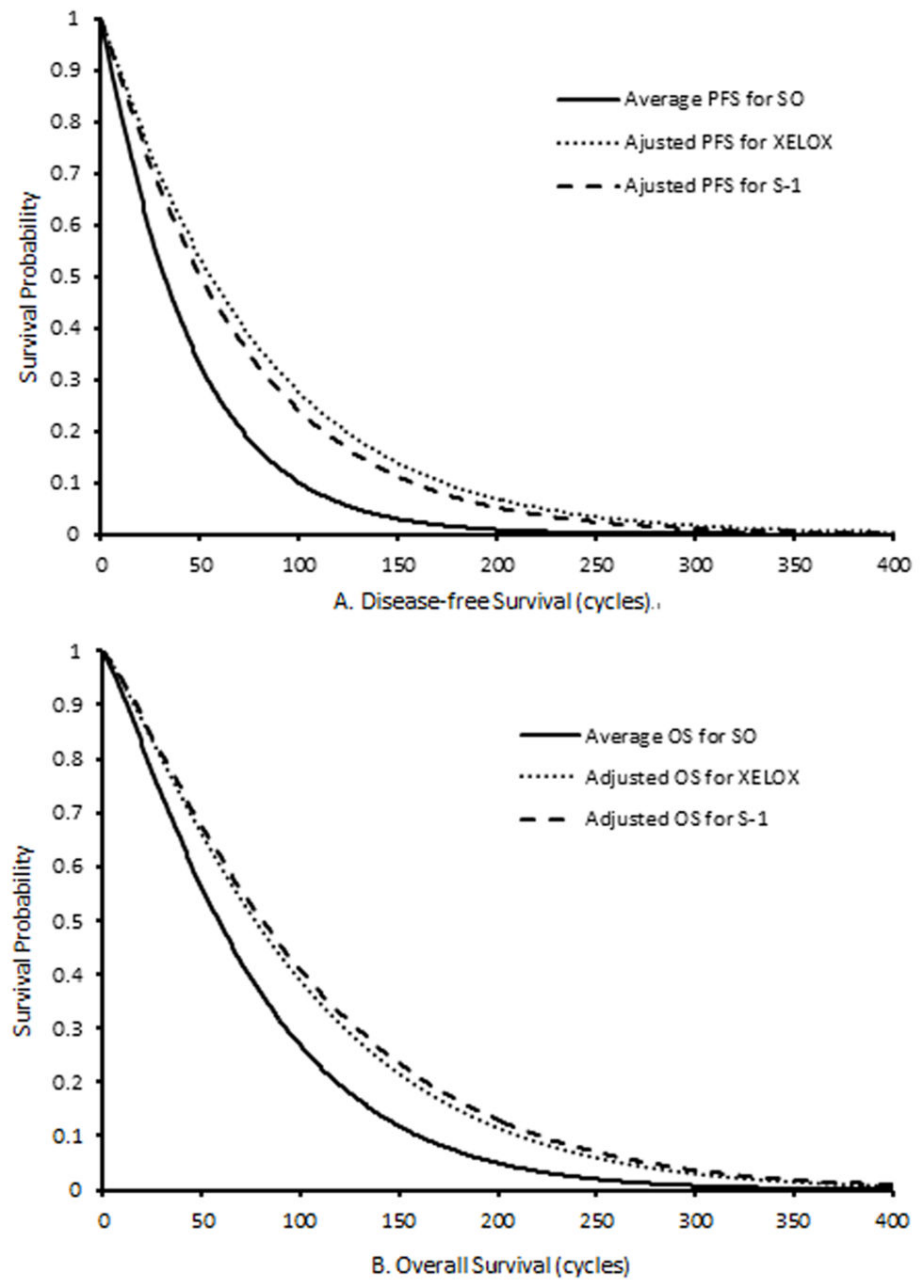
The Weibull curves of (A) disease-free survival and (B) overall survival.

The Weibull survival curves were fitted to the number of patients in the three states over time. The Weibull parameters, which are the hypothetical scale (λ) and shape (γ) of reference, are shown in Table 3. According to the adjusted formula, S_active strategies_ = (S_reference_)^HR^ (where S is the survival rate and HR is the hazard ratio), and λ values for the XELOX and S-1 groups were calculated as λ of the SO group multiplied by the corresponding hazard ratio, and γ values for the XELOX and S-1 groups were equivalent to γ for the SO group [21,22]. The final adjusted Weibull survival curves for PFS and OS for the three strategies are shown in Figure 2.

**Table 3:** Weibull parameters for progress-free survival (PFS) and overall survival (OS) for three strategies.

	**Scale(λ)**	**Shape(γ)**
PFS		
SO (reference)	0.017869	1.055532
XELOX	0.010007	1.055532
S-1	0.011079	1.055532
OS		
SO (reference)	0.005483	1.19
XELOX	0.003948	1.19
S-1	0.003728	1.19

SO = surgery-only; XELOX = capecitabine plus oxaliplatin.

### Resource cost data

The costs of each arms were estimated from the perspective of Chinese healthcare system, including costs of first-line and second-line strategies, follow-up and main adverse events (Table 4). The cost data for the DFS state of the three treatments were based on the two trials. We assumed that the administration of XELOX and S-1 regimens, the follow-up procedures and the probabilities of adverse events (Grade 3 or 4) were the same as those in the trials. In accordance with the two trials, the cost of follow-up in the model was assumed to include only the costs of examinations. If the gastric cancer progressed, 4-week cycles of intravenous paclitaxel (80mg/m^2^ administered 3 times per week) were administered to nearly 25% of the patients as second-line chemotherapy. Supportive care was administered to the remaining patients before death [23,25].

**Table 4:** Baseline costs, utility values and risks in three groups for patients with stage Ⅱ-ⅢB gastric cancer in China.

**Parameters**	**Median**	**Range**	**Distribution**	**Reference (s)**
**Costs**, $				
Capecitabine per 500mg	6.3	5.04-6.3	Lognormal	21
Oxaliplatin per 50mg	232.8	50.4-688.7	Lognormal	24
Paclitaxel per 30mg	115.4	67.9-234.9	Lognormal	21
S-1 per 20mg	8.6	4.3-12.9	Lognormal	17
Laboratory evaluations per episode	87.6	35.2-182.7	Lognormal	22
Administration per episode	18.5	15-23	Lognormal	21
Supportive care per 3 weeks	1415.4	1022.8-2021.5	Lognormal	2
Abdominal CT per episode ^a^	105.2	52.6-157.8	Gamma	17
Abdominal MRI per episode ^a^	140.8	70.4-211.2	Gamma	17
Chest radiograph per episode ^a^	8.4	4.2-12.6	Gamma	17
**Expenditures on main adverse events(Grade 3 or 4), $**				
Nausea and vomiting per episode	39.6	17.9-76.5	Lognormal	22
Neutropenia per episode	530.8	198.5-863.1	Lognormal	21
Decreased appetite, Anorexia, Fatigue and Asthenia per episode	115.4	103.8-126.9	Lognormal	19
Diarrhoea and abdominal pain per episode	44.3	28.5-54.6	Lognormal	21
Thrombocytopenia per episode	3551.7	3156.8-3980.2	Lognormal	21
**Risk for main adverse events in XELOX arm (Grade 3 or 4)^b^**				
Nausea and vomiting	0.15	0.12-0.18	Beta	16
Neutropenia	0.22	0.176-0.264	Beta	16
Decreased appetite, Fatigue and Asthenia	0.12	0.096-0.144	Beta	16
Diarrhoea and abdominal pain	0.04	0.032-0.048	Beta	16
Thrombocytopenia	0.08	0.064-0.096	Beta	16
**Risk for main adverse events in S-1 arm(Grade 3 or 4)^b^**				
Nausea	0.037	0.0296-0.0444	Beta	3
Diarrhoea	0.031	0.0248-0.0372	Beta	3
Anorexia	0.06	0.048-0.072	Beta	3
**Risk for main adverse events in SO arm (Grade 3 or 4)**	-	-	-	3, 16
**Risk for requiring second-line chemotherapy^b^**	0.25	0.2-0.3	Beta	23
**Utility^b^**				
1-5 years in DFS for XELOX arm	0.68	0.56-0.76	Beta	27
5-10 years in DFS for XELOX arm	0.81	0.648-0.972	Beta	26
1-10 years in DFS for S-1 arm	0.81	0.648-0.972	Beta	26
1-10 years in DFS for SO arm	0.81	0.648-0.972	Beta	26
Beyond 10 years for 3 arms	1			
PS in three arms	0.5	0.4-0.6	Beta	27

MRI = magnetic resonance imaging; CT = computed tomography; SO = surgery only; XELOX = capecitabine and oxaliplatin; DFS = disease-free survival; PS = progression survival.

^a^ The range was assumed to be varied ± 50%.

^b^ The range was assumed to be varied ± 20%

The costs of paclitaxel, capecitabine, administration, supportive care, neutropenia, thrombocytopenia, diarrhoea and abdominal pain were based on the report by Bin Wu [23], who calculated the costs of gastric cancer for a cost-effectiveness evaluation also in the Chinese context. Costs estimated for managing nausea and vomiting, and laboratory evaluations were from the report by Liubao Peng [24], in which an economic evaluation of adjuvant therapy for operable breast cancer was performed under the Chinese healthcare perspective. The cost of oxaliplatin per 50 mg and S-1 per 20 mg were estimated using the prices of different brands and the prevalence of the use of each brand in China [17,26]. The costs of follow up for XELOX group, including an abdominal magnetic resonance imaging (MRI) or computed tomography (CT) (every 6 months in years 1-3; yearly after 3 years) and chest radiography (every 3 months in years 1-2; every 6 months in year 3; yearly after 3 years), and the costs of follow up for S-1 group, including CT (every 6 months in years 1-2; yearly after 2 years) and laboratory evaluations ( every 2 months in first year), were obtained from local health system [17]. Some costs associated with adverse events in China have not been reported in the published literature. To facilitate the calculations, the episode of cost that represented the cost of each examination or the cost of each healthcare-related event was used in this model. We assumed that the costs associated with fatigue, anorexia and asthenia per episode were the same as the cost of decreased appetite per episode. The cost of abdominal pain per episode was assumed to be the same as the cost of diarrhoea per episode. The GDP data that we obtained were the national accounts in 2010, which were calculated at current prices, from China Statistics Press [27]. Therefore, the 2010 exchange rate from RMB (¥) to USD ($), rather than the 2012 exchange rate, was used in this study ($1=¥6.6515) [28].

### Utilities

The utility values represented the health-related quality of life for each health state. The values were based on previously published studies about health states similar to the states of the patients enrolled in the two trials. For resected gastric cancer patients, the utility of the post-gastrectomy state was estimated at 0.81 by Gockel et al. [29], who assessed quality of life using the Gastrointestinal Life Quality Index (GLQI) in 338 patients who underwent gastric resection. This utility was calculated by 116/144 (116 was the score for patients with gastrectomy and 144 was the total score of GLQI); the utilities of DFS received intravenous chemotherapy and terminal care were 0.68 and 0.50 respectively, according to Sakamaki et al. [30], who assessed utilities using Time Trade-Off (TTO) in patients with gastric cancer, the way for administration of which was the same as our study. In our study, the model tracked with lifetime, we assumed that the quality of life for patients between 5 and 10 years post-treatment was identical across the three regimens; the quality of life for patients beyond 10 years was equal to the quality of life among normal populations. Therefore we assumed the utility values were 0.68 (1-5 years), 0.81 (5-10 years) and 1.0 (beyond 10 years) for the XELOX group in the DFS stage; 0.81 (1-10 years) and 1.0 (beyond 10 years) for the S-1 and SO groups in the DFS stage; and 0.5 for individuals in PS stage in any of the three arms (as shown in Table 4).

### Sensitivity analysis

We performed a one-way sensitivity analysis for 29 variables and created a tornado diagram of the incremental cost-effectiveness ratios (ICER). A probabilistic sensitivity analysis using 1,000 Monte Carlo repetitions was performed to assess the influence of parameter uncertainties on the results of the model. The median, range, and distribution of each parameter (based on information from the literature and from the local health care system) are shown in Table 3. In accordance with China’s Guidelines for Pharmacoeconomic Evaluations (2011) [18], the discount rate in this analysis was assumed to range from 0% to 8% and the WTP threshold was set at three times the Chinese per capita GDP ($13,527) in 2010 [27]. 

## Results

**Figure 3:**
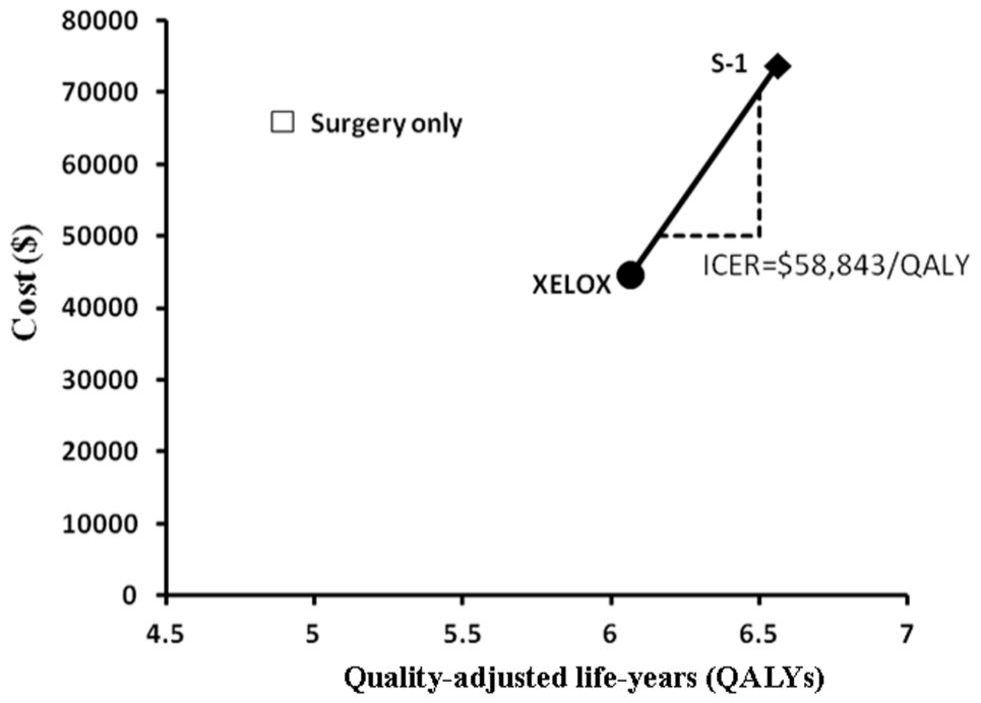
Base-case results on the cost-effectiveness of first-line strategies for resectable gastric cancer patients. The line joining strategies of capecitabine and oxaliplatin (XELOX) and S-1 is the ‘efficiency frontier’. Strategy above the line is dominated. In the cost-effectiveness plane, the value of incremental cost-effectiveness ratios (ICER) is depicted.

When the model tracked 30 years, patients of 95.7% in S-1 arm, 97.0% in XELOX arm and 98.9% in SO arm were in the state of death. The base case results with a lifetime horizon are displayed in Table 5. The cost per QALY gained was the primary outcome of the analysis. The QALYs achieved with the XELOX (5.56 QALYs) and S-1 (5.57 QALYs) strategies in the DFS state resulted in longer adjusted survival times than those achieved with SO therapy (3.75 QALYs). The total cost ($44,658) and the cost-effectiveness ratio (CER, $7,360/QALY) for XELOX were the lowest of the three treatments, as shown in Table 5. The relative scenario analyses, as shown in Figure 3, suggested that the SO strategy was inferior to the other options. Compared to XELOX, S-1 was showed an incremental gain of 0.49 QALYs at an additional cost of $29,058, which suggested that ICER of S-1 was $58,843 per QALY (Table 5).

**Table 5:** The base-case results for three treatments.

**Model outcome**	**Treatment strategy**		
	**SO**	**XELOX**	**S-1**
Costs in DFS($)	954	15,555	17,563
Costs in PS($)	64,940	29,102	56,153
Costs of total($)	65,894	44,658	73,716
QALYs in DFS(QALY)	3.75	5.56	5.57
QALYs in PS(QALY)	1.14	0.51	0.99
QALYs of total(QALY)	4.89	6.07	6.56
CER($/QALY)	13,468	7,360	11,235
ICER for XELOX ($/QALY)	dominated	-	58,843
ICER for SO($/QALY)	-	dominant	4,688

DFS = Disease-free survival; PS = progression survival; QALYs = quality-adjusted life-year; SO = surgery only; XELOX = capecitabine and oxaliplatin; CER = cost-effectiveness ratio; ICER = incremental cost-effectiveness ratio.

**Figure 4:**
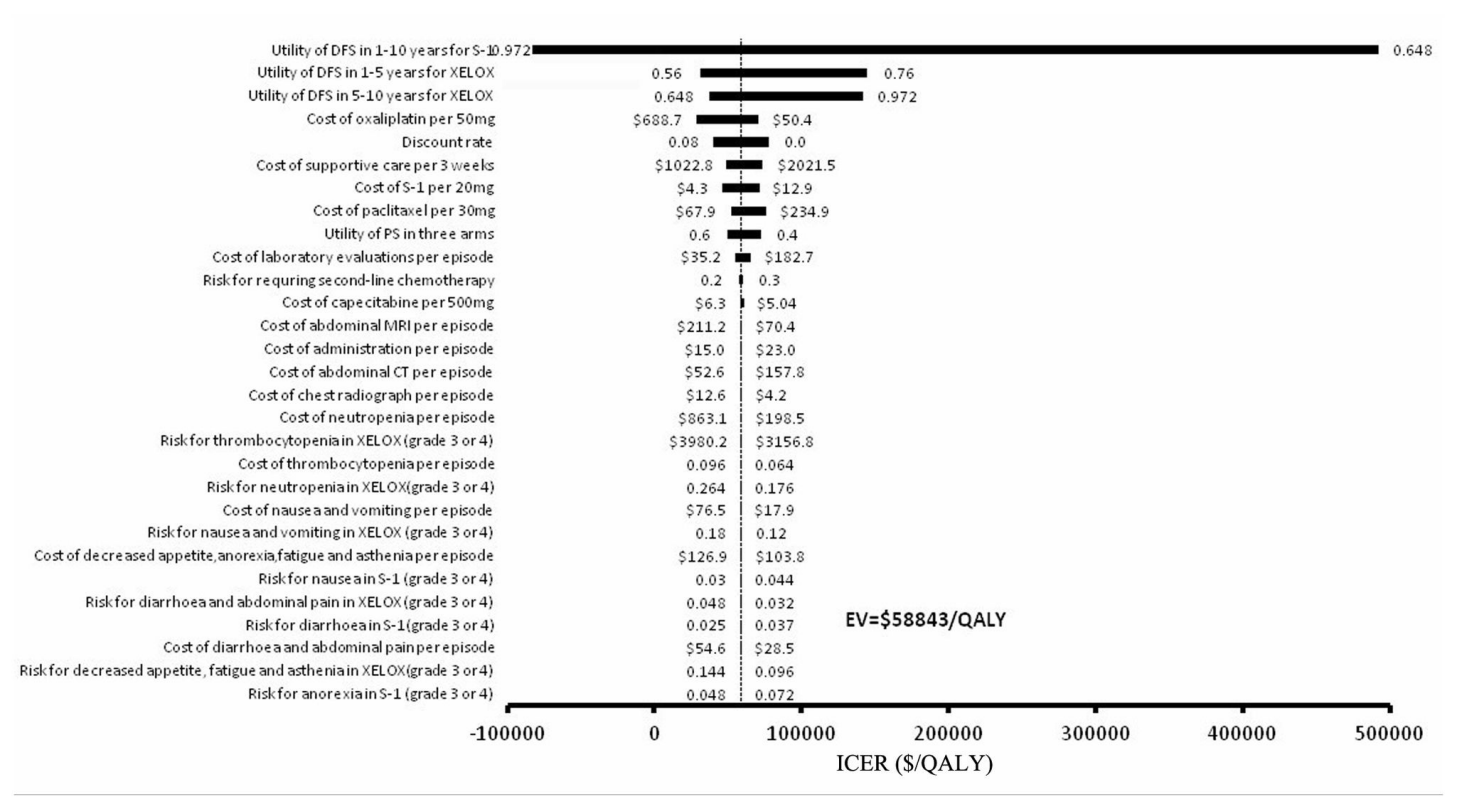
Tornado diagram for one-way sensitivity analysis of the capecitabine plus oxaliplatin and the S-1 treatment strategies. The parameters tested in the one-way sensitivity analysis are shown in the y-axis. The vertical dashed line represents $58,843/QALY (the results of base-case).

The tornado diagram (Figure 4) shows the one-way sensitivity analysis for the ICER, demonstrating the varying incremental values between the XELOX and the S-1 treatment strategies. In the model, the utility of DFS for 1 to 10 years after S-1 treatment was the most influential parameter (ranging from 0.648 to 0.972, with the ICER increasing from $-82,561 per QALY to $409,654 per QALY). The other two most influential variables included the utility of DFS for 1-5 years after XELOX treatment and the utility of DFS for 5-10 years after XELOX. The least influential parameters were the costs of adverse events, such as decreased appetite, fatigue and asthenia with XELOX treatment (Grade 3 or 4), the cost of diarrhoea and abdominal pain per episode, and the risk for anorexia with S-1 treatment (Grade 3 or 4).

**Figure 5:**
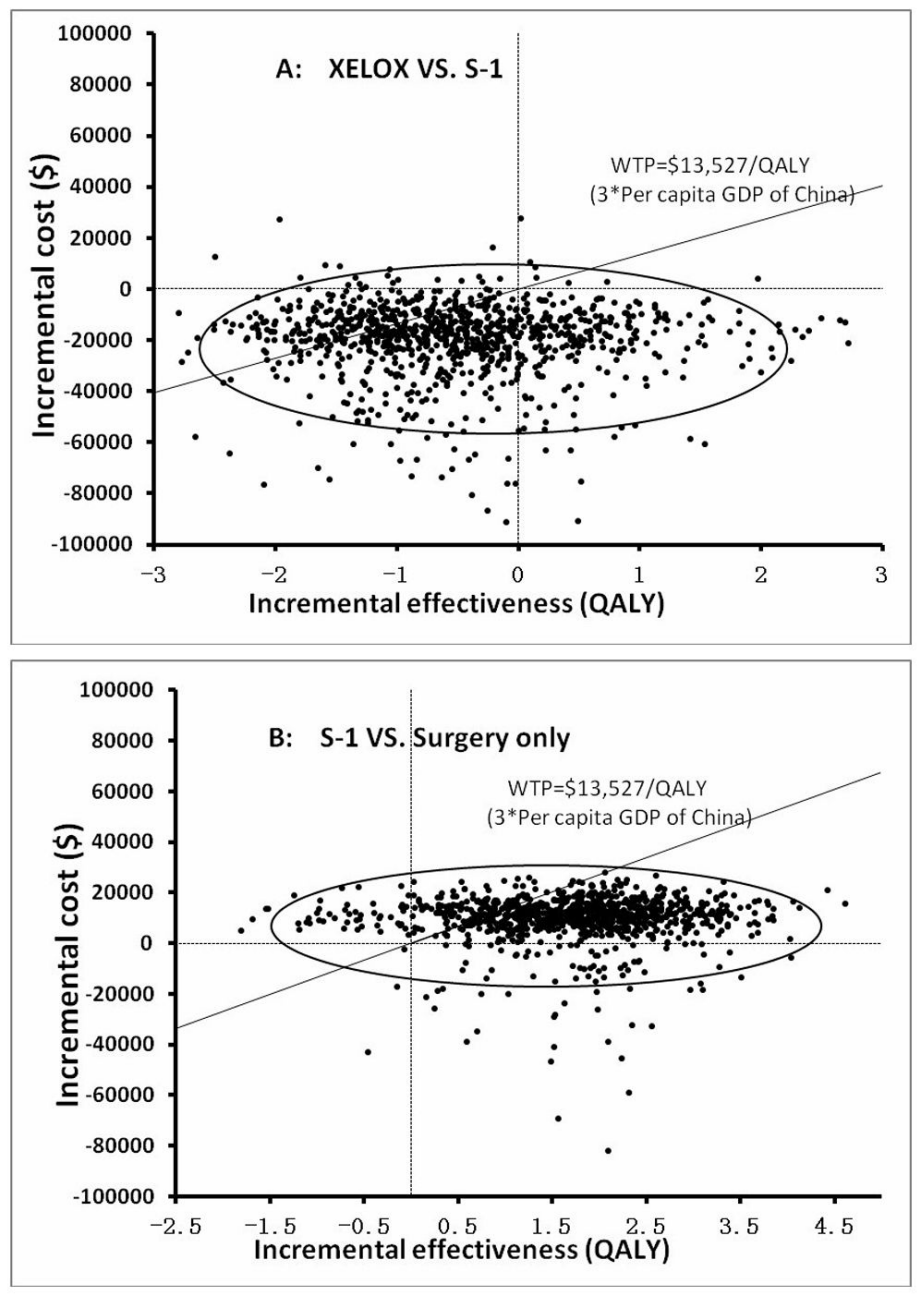
The results of Monte Carlo probabilistic sensitivity analysis for the strategies of capecitabine and oxaliplatin (XELOX) VS. S-1 (A) and S-1 VS. surgery only (B) are shown in two scatter plots. The solid lines indicate the $13,527 threshold. The estimates of 95% were surrounded in the ellipses.

The ICER scatterplot (Figure 5) shows the results of the probabilistic sensitivity analysis with a threshold of $13,527, including a set of points that represent pairs of incremental cost and effectiveness values from the Monte Carlo simulation (1,000 repetitions). The estimates of 95% were surrounded in the ellipses. As Figure 5A reflected, plots under the threshold line account for 75.8% of all scatterplot, which indicates that compared to the S-1 group, the probability that the treatment of XELOX was cost-effective was 75.8%. In comparison, the Figure 5B indicates that compared to the SO strategy, the probability that S-1 was cost-effective was 81.8%..

**Figure 6:**
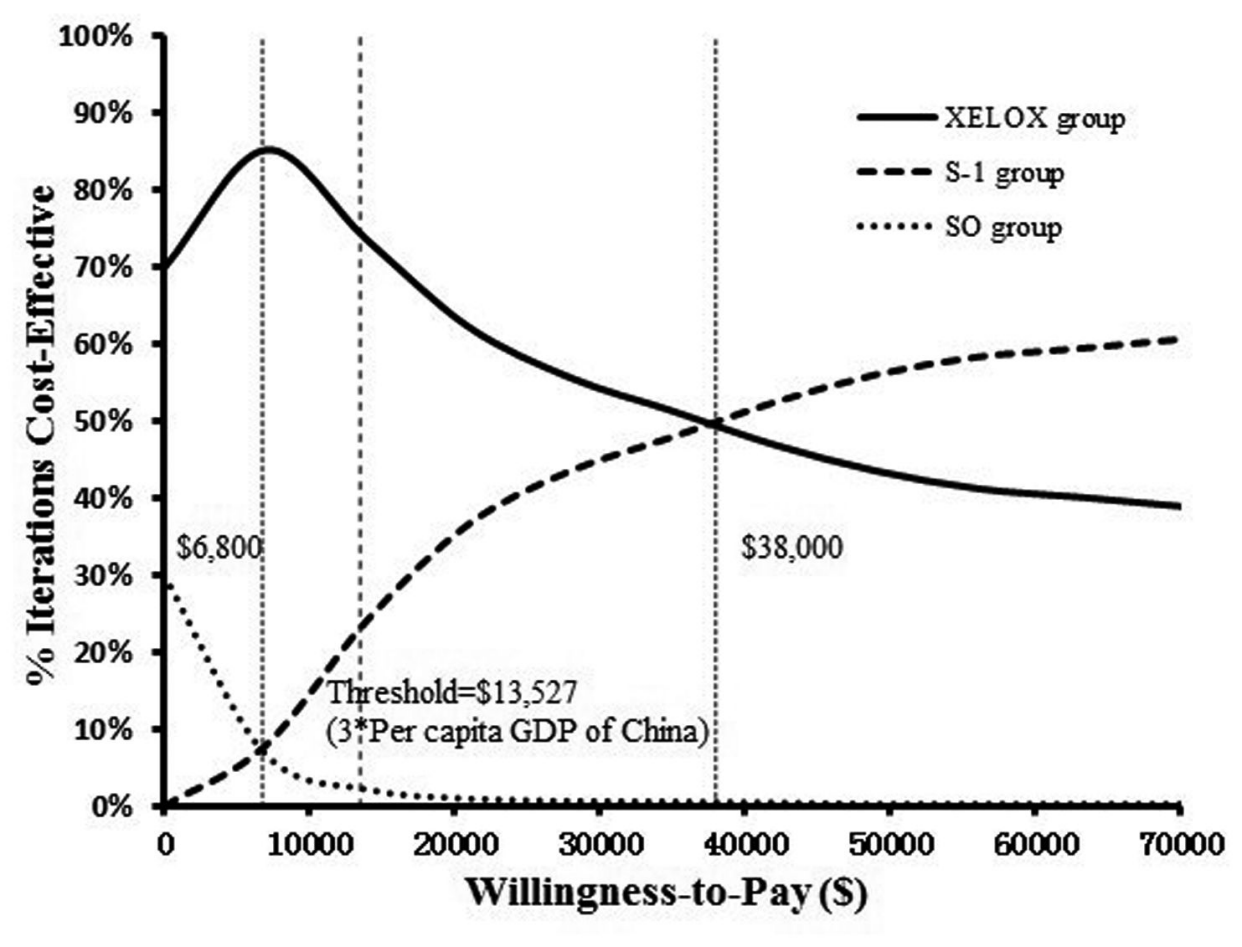
Acceptability curves for the choice of three treatment strategies at different willingness-to-pay (WTP) thresholds in Chinese resectable gastric cancer patients. Three vertical dashed lines represent different WTP thresholds.

The acceptability curve (Figure 6) shows the changing percentage of cases, generated by simulation, for which treatment with XELOX was more cost-effective than the other strategies when the threshold was less than $38,000. The relative cost-effectiveness changed as a function of the WTP threshold. When the WTP was $13,527 for each QALY gained, the probability was nearly 73% that the XELOX treatment was cost-effective. When the threshold was more than $38,000, the likelihood of cost-effectiveness achieved by the S-1 group was greater than 50%, followed by the XELOX group (less than 50%) and the SO group (0%).

## Discussion

Before 2010, no adjuvant chemotherapy after D2 gastrectomy was included in the Chinese NCCN Clinical Practice Guidelines in Oncology: Gastric Cancer. Fortunately, as medical science progressed, the S-1 regimen was recommended in 2010, and the XELOX strategy was added in 2011. The two regimens can appreciably improve DFS and OS. However, China has a large population (1.3 billion) with differing degrees of education and a large disparity between the rich and poor. After surgery, some resectable gastric cancer patients would not choose the two recommended strategies; instead, they would forego further treatment or pursue traditional Chinese treatment if they had no pain in the short term [31-33]. The growing cost of treatment for gastric cancer has become a serious economic burden for society, families, and patients in China [34]. Therefore, studying the economic implications of the three strategies is important for the fields of medicine and policy.

In the base case, compared with XELOX, S-1 increased QALYs by 0.58 and increased costs by $29,058 per patient; SO reduced QALYs by 1.18 and increased costs by $21,236 per patient. The resulting ICERs ($58,843/QALY and $-18,077/QALY, respectively) indicate that neither S-1 nor SO is cost-effective based on the threshold of $13,527 (three times the Chinese per capita GDP). Compared to SO, XELOX improved QALYs by 1.18 and decreased costs by $18,077, which indicates that XELOX is cost-effective. S-1 improved QALYs by 1.82 and increased costs by $7,822, and the resulting ICER of $4,688/QALY indicates that S-1 is cost-effective based on the threshold of $13,527. Therefore, XELOX as a first-line adjuvant chemotherapy for resectable gastric cancer patients in China provided more health and economic benefits than the SO and S-1 strategies, given a WTP threshold of $13,527.

Three tornado diagrams were created with TreeAge Pro software to compare the three therapies. We found that the most sensitive parameters were the utilities of the three regimens, and the least sensitive parameters were the costs of adverse events. Therefore, we only show the tornado diagram (Figure 4) for XELOX vs. S-1, which is the most important comparison for healthcare providers, patients and policymakers. The diagram shows that enhancing quality of life and decreasing the price of pharmaceuticals is the ideal combination for some new regimens.

Our probabilistic sensitivity analysis showed that the cost-effectiveness results changed as the WTP thresholds were modified (as shown in Figure 6). On average, three times the Chinese per capita GDP is $13,527. However, the per capita GDP differs significantly among the 32 provinces of the Chinese mainland. The maximum difference is $8,875 ($10,828 per capita GDP in Shanghai; $1,953 per capita GDP in Guizhou [21]). For all of the provinces, three times the per capita GDP was less than $38,000 [21]. Therefore, XELOX is the most cost-effective, first-line, adjuvant chemotherapy for resectable gastric cancer patients on the Chinese mainland, followed by S-1.

Several limitations apply to the current study. An inevitable limitation of this process was the use of a Weibull distribution to extrapolate the results beyond the lifetime horizon of the trial. Another limitation was that our study did not account for some necessary costs that were not mentioned in the CLASSIC trial, such as hospitalisation expenses resulting chemotherapy, in some patients who took XELOX. The last important limitation was the absence of head-to-head trial for the three strategies. However, striking parallels of the CLASSIC and ACTS-GC trials, such as the study design, eligibility criteria and control group composition, could make up some shortfall.

## Conclusions

Our results suggest that for patients in China with resectable disease, first-line adjuvant chemotherapy with XELOX after a D2 gastrectomy is a best option comparing with S-1 and SO in view of our current study. In addition, S-1 might be a better choice, especially with a higher value of WTP threshold. 

## References

[1] International Agency for Research on Cancer (2008) Stomach Cancer Incidence, Mortality and Prevalence Worldwide in 2008 Summary. Available: http://globacan.iarc.fr/factsheets/cancers/stomach.asp. Accessed 2012 July 10

[2] GASTRIC (Global Advanced/Adjuvant Stomach Tumor Research International Collaboration) Group, Paoletti X, Oba K, Burzykowski T, Michiels S et al. (2010) Benefit of adjuvant chemotherapy for resectable gastric cancer: a meta-analysis. JAMA 303: 1729–1737 10.1001/jama.2010.534 20442389

[3] Sakuramoto S, Sasako M, Yamaguchi T, Kinoshita T, Fujii M et al. (2007) Adjuvant chemotherapy for gastric cancer with S-1, an oral fluoropyrimidine. N Engl J Med 357: 1810–1820. doi:10.1056/NEJMoa072252. PubMed: 17978289. 17978289

[4] NCCN clinical practice guidelines in Oncology-Gastric cancer guideline (2010) [in Chinese ]. Available: http://www.nfhoc.com/2010NCCN/%E8%83%83%E7%99%8C.pdf . Accessed 2012 July 10

[5] NCCN clinical practice guidelines in Oncology-Gastric cancer guideline (2011) [in Chinese ]. Available: http://www.nccnchina.org.cn/nccn-guidelines-china.aspx . Accessed 2012 July 10

[6] Ajani JA, Lee FC, Singh DA, Haller DG, Lenz HJ et al. (2006) Multicenter Phase II trial of S-1 plus cisplatin in patients with untreated advanced gastric or gastroesophageal junction adenocarcinoma. J Clin Oncol 24: 663–667. doi:10.1200/JCO.2005.04.2994. PubMed: 16446338. 16446338

[7] Boku N, Yamamoto S, Fukuda H, Shirao K, Sawaki A et al. (2009) Fluorouracil versus combination of irinotecan plus cisplatin versus S-1 in metastatic gastric cancer: a randomised Phase 3 study. Lancet Oncol 10: 1063–1069. doi:10.1016/S1470-2045(09)70259-1. PubMed: 19818685. 19818685

[8] Kubota T (2008) The role of S-1 in the treatment of gastric cancer. Br J Cancer 98: 1301–1304. doi:10.1038/sj.bjc.6604332. PubMed: 18362933. 18362933 PMC2361710

[9] Koizumi W, Narahara H, Hara T, Takagane A, Akiya T et al. (2008) S-1 plus cisplatin versus S-1 alone for first-line treatment of advanced gastric cancer (SPIRITS trial): a Phase III trial. Lancet Oncol 9: 215–221. doi:10.1016/S1470-2045(08)70035-4. PubMed: 18282805. 18282805

[10] Sasako M, Sakuramoto S, Katai H, Kinoshita T, Furukawa H et al. (2011) Five-Year outcomes of a randomized phase Ⅲ trial comparing adjuvant chemotherapy with S-1 versus surgery alone in stage Ⅱ or iii. Gastric Cancer - J Clin Oncol 29(33): 4387-4393. 22010012 10.1200/JCO.2011.36.5908

[11] Miwa M, Ura M, Nishida M, Sawada N, Ishikawa T et al. (1998) Design of a novel oral fluoropyrimidine carbamate, capecitabine, which generates 5-fluorouracil selectively in tumours by enzymes concentrated in human liver and cancer tissue. Eur J Cancer 34: 1274-1281. doi:10.1016/S0959-8049(98)00058-6. PubMed: 9849491. 9849491

[12] Gramont A, Figer A, Seymour M, Homerin M, Hmissi A et al. (2000) Leucovorin and fluorouracil with or without oxaliplatin as first-line treatment in advanced colorectal cancer. J Clin Oncol 18: 2938-2947. PubMed: 10944126. 10944126 10.1200/JCO.2000.18.16.2938

[13] Dong N, Jiang W, Li H, Liu Z, Xu X et al. (2009) Triweekly oxaliplatin plus oral capecitabine as first-line chemotherapy in elderly patients with advanced gastric cancer. Am J Clin Oncol 32: 559-563. doi:10.1097/COC.0b013e3181967db3. PubMed: 19581793. 19581793

[14] Liu C, Sun Q, Hang X, Zhong B, Wang D (2008) Multicenter phase II study of capecitabine plus oxaliplatin as a first-line therapy in Chinese patients with advanced gastric cancer. Anticancer Drugs 19: 825-831. doi:10.1097/CAD.0b013e32830c457e. PubMed: 18690095. 18690095

[15] Park YH, Lee JL, Ryoo BY, Ryu MH, Yang SH et al. (2008) Capecitabine in combination with oxaliplatin (XELOX) as a first-line therapy for advanced gastric cancer. Cancer Chemother Pharmacol 61: 623-629. doi:10.1007/s00280-007-0515-7. PubMed: 17522863. 17522863

[16] Bang Yung-Jue, Kim Young-Woo, Yang Han-Kwang, Cheol Chung Hyun, Park et al Young-Kyu. (2012) Adjuvant capecitabine and oxaliplatin for gastric cancer after D2 gastrectomy (CLASSIC): a phase 3 open-label, randomised controlled trial. Lancet 379: 315-321. doi:10.1016/S0140-6736(11)61873-4. PubMed: 22226517. 22226517

[17] http://www.hnyyjg.com.

[18] China Center for Health Economic Research.China Guidelines for Pharmacoeconomic Evaluations (Version 8) [in Chinese]. Available: http://www.ispor.org/PEguidelines/countrydet.asp?c=28&t=4. Accessed 2012 July 5

[19] Rituximab for the treatment of relapsed follicular lymphoma (2007). Available http://www.nice.org.uk/nicemedia/live/11730/38897/38897.pdf. Accessed 2013 October 1

[20] http://seer.cancer.gov/statfacts/html/stomach.html.

[21] Wu Bin, Dong Baijun, Xu Yuejuan, Zhang Qiang, Jinfang Shen et al. (2012) Economic evaluation of first-line treatment for metastatic renal cell carcinoma: a cost-effectiveness analysis in a health resource-limited setting. PLOS ONE 7(3): e32530. doi:10.1371/journal.pone.0032530. PubMed: 22412884. 22412884 PMC3297611

[22] Hoyle M, Green C, Thompson-Coon J, Liu Z, Welch K et al. (2010) Cost effectiveness of temsirolimus for first line treatment of advanced renal cellcarcinoma. Value in Health 13(1): 61–68. doi:10.1111/j.1524-4733.2009.00617.x. PubMed: 19804430. 19804430

[23] Wu Bin, Ye Ming, Chen Huafeng, Shen Jinfang (2012) Costs of Trastuzumab in combination with chemotherapy for HER2-positive advanced gastric or gastroesophageal junction cancer: an economic evaluation in the Chinese context. Clin Ther 34(2): 468-479. doi:10.1016/j.clinthera.2012.01.012. PubMed: 22325735. 22325735

[24] Liubao P, Xiaomin W, Chongqing T, Karnon J, Gannong C et al. (2009) Cost-effectiveness analysis of adjuvant therapy for operable breast cancer from a Chinese perspective: doxorubicin plus cyclophosphamide versus docetaxel plus cyclophosphamide. Pharmacoeconomics 27(10): 873-886. doi:10.2165/11314750-000000000-00000. PubMed: 19803541. 19803541

[25] Wilson D, Hiller L, GehJi (2005) Review of second-line chemotherapy for advanced gastric adenocarcinoma. Clin Oncol (R Coll Radiol) 17: 81-89. doi:10.1016/j.clon.2004.10.006. PubMed: 15830569. 15830569

[26] Guangzhou (2010) Biaodian Medical information co. LTD Report on antitumor (anticancer) drug market in 2010 [in Chinese]. Available: http://wenku.baidu.com/view/370ed272f242336c1eb95e22.html . Accessed 2012 July 2

[27] Gross Domestic Product (2010). Available: http://www.stats.gov.cn/tjsj/ndsj/2011/html/C0201e.htm. Accessed 2012 July 6

[28] The People’s Bank of China (2010). Available: http://www.pbc.gov.cn. Accessed 2012 July 17

[29] Gockel I, Pietzka S, Junginger T (2005) Quality of life after subtotal resection and gastrectomy for gastric cancer. Chirurg 76: 250-257. doi:10.1007/s00104-004-0950-5. PubMed: 15551010. 15551010

[30] Sakamaki H, Ikeda S, Yajima S, Ikegami N, Tanaka K et al. (2009) Cost-utility analysis of the oral Fluoropyrimidine S-1 Versus conventional intravenous chemotherapy in advanced or recurrent gastric cancer. Open Health Services and Policy Journal 2: 26-33. doi:10.2174/1874924000902010026.

[31] Zong-pei Tong, Qi-lin Zhou, Cui-xiang Xu. (2011) Early enteral nutrition combined with traditional Chinese Medicine for postoperative gastric carcinoma: a report of 12 cases. Journal of Anhui TCM College 30(2): 30-32.

[32] Wen Chen, ShuPeng (2011) The research status of treating gastric cancer with traditional Chinese Medicine of strengthening spleen and warding off disaster. Gansu Journal of TCM 24(4): 76-78.

[33] He Ping Shen.Ke-ping, Hu Bing. (2012) Progress of traditional Chinese Medicine treating gastric cancer. Chinese archives of traditional Chinese Medicine 30(2): 280-282

[34] Xiao-le Cun, Xiang-xian Feng, Shao-xia Li (2010) Analysis of hospitalization expenditures among 3287 gastric carcinoma inpatients in general hospital. Chin J Public Health 26(6): 703-704.

